# Risk Factors for Tracheobronchomalacia in Preterm Infants With Bronchopulmonary Dysplasia

**DOI:** 10.3389/fped.2021.697470

**Published:** 2021-06-25

**Authors:** Ya-Ting Su, Chun-Che Chiu, Shen-Hao Lai, Shao-Hsuan Hsia, Jainn-Jim Lin, Oi-Wa Chan, Chih-Yung Chiu, Pei-Ling Tseng, En-Pei Lee

**Affiliations:** ^1^Chang Gung University College of Medicine, Taoyuan, Taiwan; ^2^Division of Pediatric Endocrinology and Genetics, Department of Pediatrics, Chang Gung Memorial Hospital, Chang Gung University College of Medicine, Taoyuan, Taiwan; ^3^Department of Pediatrics, Tucheng Composite Municipal Hospital, New Taipei City, Taiwan; ^4^Division of Pediatric Pulmonology, Department of Pediatrics, Chang Gung Memorial Hospital at Linkou, Taoyuan, Taiwan; ^5^Division of Pediatric Critical Care Medicine, Department of Pediatrics, Chang Gung Memorial Hospital at Linkou, Taoyuan, Taiwan; ^6^College of Nursing, Taipei Medical University, Taipei, Taiwan

**Keywords:** preterm infants, tracheobronchomalacia, bronchopulmonary dysplasia, bronchoscopy, risk factors

## Abstract

**Aim:** To identify the risk factors associated with the development of tracheobronchomalacia (TBM) in preterm infants with bronchopulmonary dysplasia (BPD).

**Methods:** This was a retrospective cohort study using chart reviews of preterm infants born at ≤ 36 week's gestation who underwent flexible fiberoptic bronchoscopy in a tertiary pediatric referral center between January 2015 and January 2020. Indications for the bronchoscopy examination included lobar atelectasis on plain chest film, persistent CO_2_ retention, recurrent extubation failure, or abnormal breathing sounds such as wheeze or stridor. Optimal cutoff values for each risk factor were also determined.

**Results:** Fifty-eight preterm infants with BPD were enrolled, of whom 29 (50%) had TBM. There were no significant differences in gestational age and birth weight between those with and without TBM. Significantly more of the patients with TBM had severe BPD compared to those without TBM (68.9 vs. 20.6%, *p* < 0.001). Clinical parameters that were significantly different between the two groups were included in multivariate analysis. Among these factors, severe BPD was the most powerful risk factor for the development of TBM (odds ratio 5.57, 95% confidence interval 1.32–23.5, *p* = 0.019). The areas under the receiver operating characteristic curves for peak inspiratory pressure (PIP) and the duration of intubation were 0.788 and 0.75, respectively. The best predictive cutoff values of PIP and duration of intubation for TBM were 18.5 mmHg and 82 days, respectively.

**Conclusion:** Preterm infants with severe BPD are at high risk for the development of TBM, and the risk is even higher in those who receive a higher PIP or are intubated for longer. Bronchoscopy examinations should be considered for the early diagnosis and management of TBM in infants with these risk factors.

## Introduction

Tracheobronchomalacia (TBM) is defined as weak and soft muscular walls and cartilage of the airways. Children with TBM may have an increase in airway compliance and excessive dynamic collapse of the airway during the respiratory cycle which can result in clinical symptoms (1). There are two kinds of TBM; extrathoracic and intrathoracic. Extrathoracic (cervical) TBM is less common in children, and causes inspiratory symptoms such as stridor, a prolonged inspiratory period, and lower lung volume. In comparison, intrathoracic TBM is more common in children and can cause expiratory symptoms such as wheezing, a prolonged expiratory period, and difficultly clearing secretions. Therefore, pediatric intrathoracic TBM is usually accompanied by recurrent lower respiratory tract illnesses, difficulty in weaning from a ventilator, and life-threatening events (2, 3).

Previous studies have demonstrated that the incidence of intrathoracic TBM in preterm infants who undergo flexible bronchoscopy is often underestimated, with reports ranging from 16 to 50% (4, 5). Airway compliance is inversely proportional to the gestational age of newborns, which means that preterm infants have more compliant airways compared to term infants. In addition, infants with bronchopulmonary dysplasia (BPD) usually use accessory muscles to exhale, which can elevate pleural pressure resulting in airway collapse (1). Moreover, preterm infants with BPD need prolonged positive pressure ventilation, which can cause airway wall injury (6). Consequently, the development of TBM is highly correlated with preterm infants with BPD (1).

Despite the association between TBM and BPD in preterm infants, few case series have reported the outcomes in these infants (5, 7, 8). Only one study conducted in 1995 analyzed the risk factors for developing TMB in preterm infants with BPD, and it reported that a younger gestational age and higher mean airway pressure (MAP) during the first week after birth were associated with TBM (5). However, comprehensive analyses of the risk factors associated with TBM in preterm infants with BPD are lacking. Therapeutic strategies to prevent the development of TBM in this population are urgently needed. Therefore, the aim of this study was to analyze the risk factors and outcomes of TBM in preterm infants with BPD.

## Materials and Methods

### Patient Population

We performed a retrospective cohort study and enrolled preterm infants born at ≤ 36 week's gestation who underwent flexible fiberoptic bronchoscopy in a tertiary pediatric referral center between January 2015 and January 2020. Indications for the bronchoscopy examinations included lobar atelectasis on plain chest film, persistent CO_2_ retention, recurrent extubation failure, or abnormal breathing sounds such as wheeze or stridor. Infants with congenital heart diseases or congenital airway malformations were excluded from the study. The need for informed consent was waived by the Chang Gung Medical Foundation Institutional Review Board. This study was approved by the Ethics Committee of Chang Gung Memorial Hospital (approval no. 202000797B0).

### Study Design

Clinical data including gestational age, birth weight, sex, and age at bronchoscopy examination were retrospectively reviewed. Average ventilator settings during the first week of life and during the week before the bronchoscopy examination were also collected. The diagnosis of BPD was based on the modified NHLBI guidelines (9). The total duration of intubation from birth to the bronchoscopy examination was recorded according to the patient's electronic medical charts.

### Definition of Tracheobronchomalacia

Endoscopic airway evaluations were performed using flexible fiberoptic bronchoscopes (BF-Q290, 2.8 mm outer diameter; Olympus, Inc., Tokyo, Japan) by three experienced pediatric pulmonologists. All bronchoscopy images were reviewed by two authors (C.C. Chiu and E.P. Lee) who were blinded to the identity of the patients. A diagnosis of TBM was confirmed when a >50% reduction in the airway cross-sectional area on expiration was observed under direct bronchoscopy inspection (10, 11).

### Outcomes

The main outcomes for this study were extubation failure (times), length of stay, duration of mechanical ventilation, death, tracheostomy, gastrostomy, and a physician diagnosis of pneumonia, gastroesophageal reflux disease (GERD) in the medical record during the ICU hospitalization.

### Statistical Analysis

Data were reported as mean ± standard deviation (SD) or median (interquartile range). Univariate analyses were performed using the chi-square test, Fisher's exact test or Mann-Whitney U test, as appropriate. Multivariate logistic regression analysis with stepwise selection was conducted to identify independent predictors of TBM. Finally, receiver operating characteristic (ROC) curve analysis was used to define the optimal cutoff values for the clinical parameters that may contribute to the development of TBM. A *P*-value <0.05 was considered to be statistically significant. IBM SPSS Statistics software (version 20.0; SPSS Statistics for Windows, Armonk, NY, USA) was used for all statistical calculations.

## Results

### Patient Demographics

**Figure 1:**
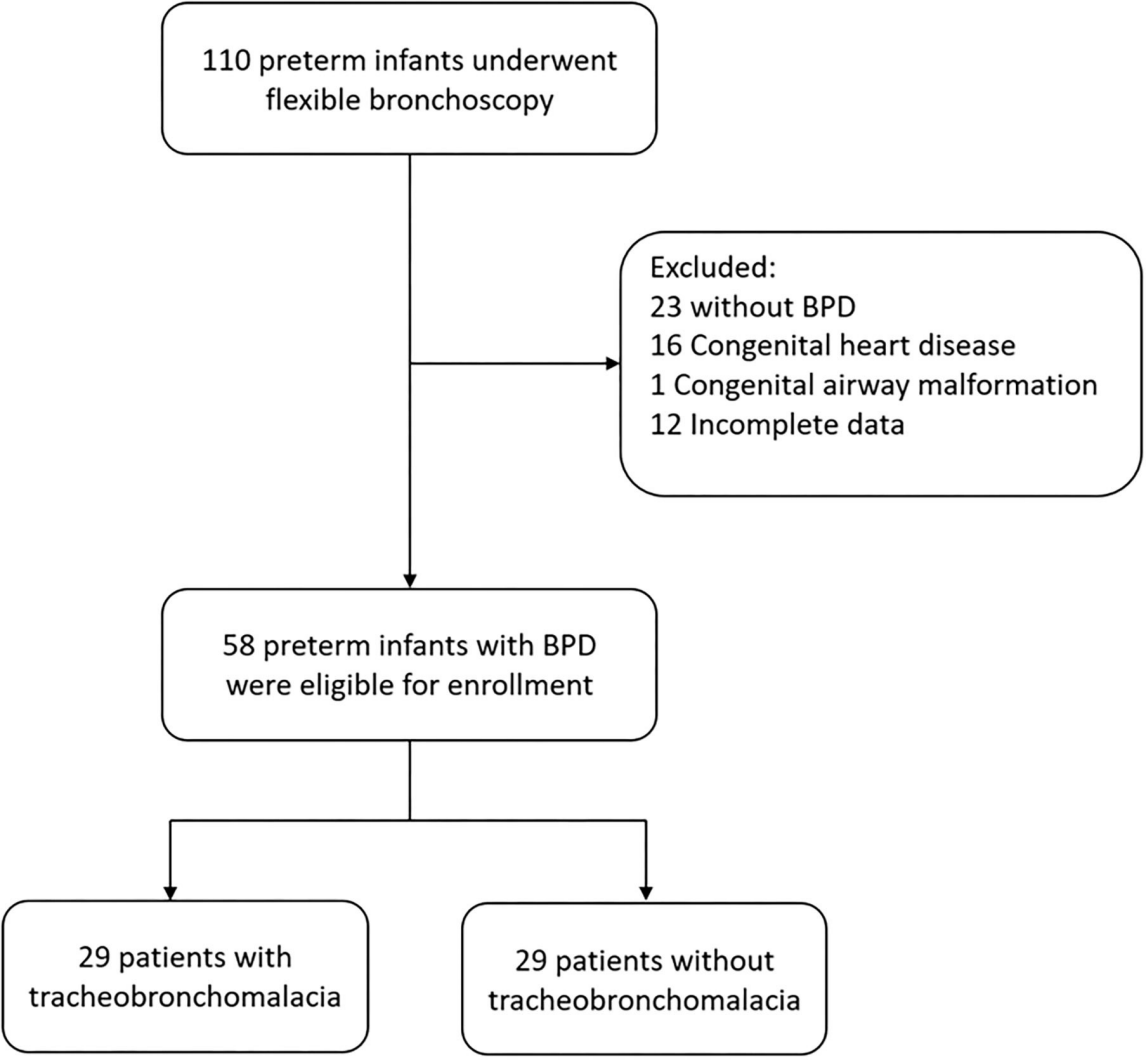
Flow diagram of study inclusion of preterm infants with BPD who underwent flexible bronchoscopy.

A total of 110 preterm infants (born at ≤ 36 week's gestation) underwent flexible bronchoscopy during the study period (Figure 1). We excluded 23 patients without BPD, 16 with complex congenital heart diseases, 1 with a congenital airway malformation (type IV laryngeal cleft), and 12 with incomplete medical records. The remaining 58 preterm infants with BPD were enrolled into the study, of whom 29 (50%) had TBM. There were no significant differences in gestational age and birth weight between those with and without TBM. None of the infants with stridor as an indication for bronchoscopy had TBM. Significantly more of the patients with TBM had severe BPD compared to those without TBM (68.9 vs. 20.6%, *p* < 0.001).

### Parameters of Mechanical Ventilation

There was no significant difference in MAP during the first week of life between the two groups (Table 1). In the week before the bronchoscopy examination, the patients with TBM were supported by higher peak inspiratory pressure (PIP), positive end expiratory pressure (PEEP), and MAP. Furthermore, the patients with TBM were intubated for longer than those without TBM.

**Table 1:** Demographic data of the preterm infants with BPD who underwent flexible bronchoscopy.

**Demographics**	**TBM (*n* = 29)**	**No TBM (*n* = 29)**	***P*-value**
Gestational age (weeks), mean (SD)	27.5 ± 2.3	27.4 ± 2.5	0.645
Birth weight (grams), mean (SD)	1056 ± 434	1019 ± 366	0.724
Sex (male), n (%)	17 (58.6)	20 (68.9)	0.585
Indication for bronchoscopy, n (%)			0.039^*^
Extubation failure	13 (44.8)	9 (31)	
Atelectasis	7 (24.1)	9 (31)	
Stridor	0	7 (24.1)	
CO_2_ retention	5 (17.2)	3 (10.3)	
Wheezing	4 (13.7)	1 (3.4)	
Age at bronchoscopy examination (days), mean (SD)	150 ± 67	131 ± 84	0.36
Post menstrual age at time of bronchoscopy examination (weeks), mean (SD)	48.8 ± 9.3	45.9 ± 12.6	0.39
Body weight at bronchoscopy examination (kg), mean (SD)	4.1 ± 1.3	3.8 ± 1.8	0.396
Severe bronchopulmonary dysplasia, n (%)	20 (68.9)	6 (20.6)	<0.001^*^
Average MAP during the first week (cmH_2_O), mean (SD)	9 ± 1.4	8 ± 1.5	0.073
**Average ventilator setting during**			
**the week before bronchoscopy, mean (SD)**			
PIP (cmH_2_O)	21.2 ± 8.3	11.7 ± 10.2	<0.001^*^
PEEP (cmH_2_O)	6.7 ± 1.2	4.1 ± 3.1	<0.001^*^
MAP (cmH_2_O)	10 ± 2.6	5.9 ± 4.6	<0.001^*^
FiO2 (%)	31.7 ± 11.7	29.6 ± 9.8	0.098
Duration of Intubation (days), mean (SD)	101.5 ± 48.2	63.2 ± 29	<0.001^*^

*TBM, tracheobronchomalacia; PIP, peak inspiratory pressure; PEEP, positive end expiratory pressure; MAP, mean airway pressure; FiO_2_, fraction of inspired oxygen.*

* *P < 0.05 statistically significant*.

### Multivariate Analysis of Parameters

**Figure 2:**
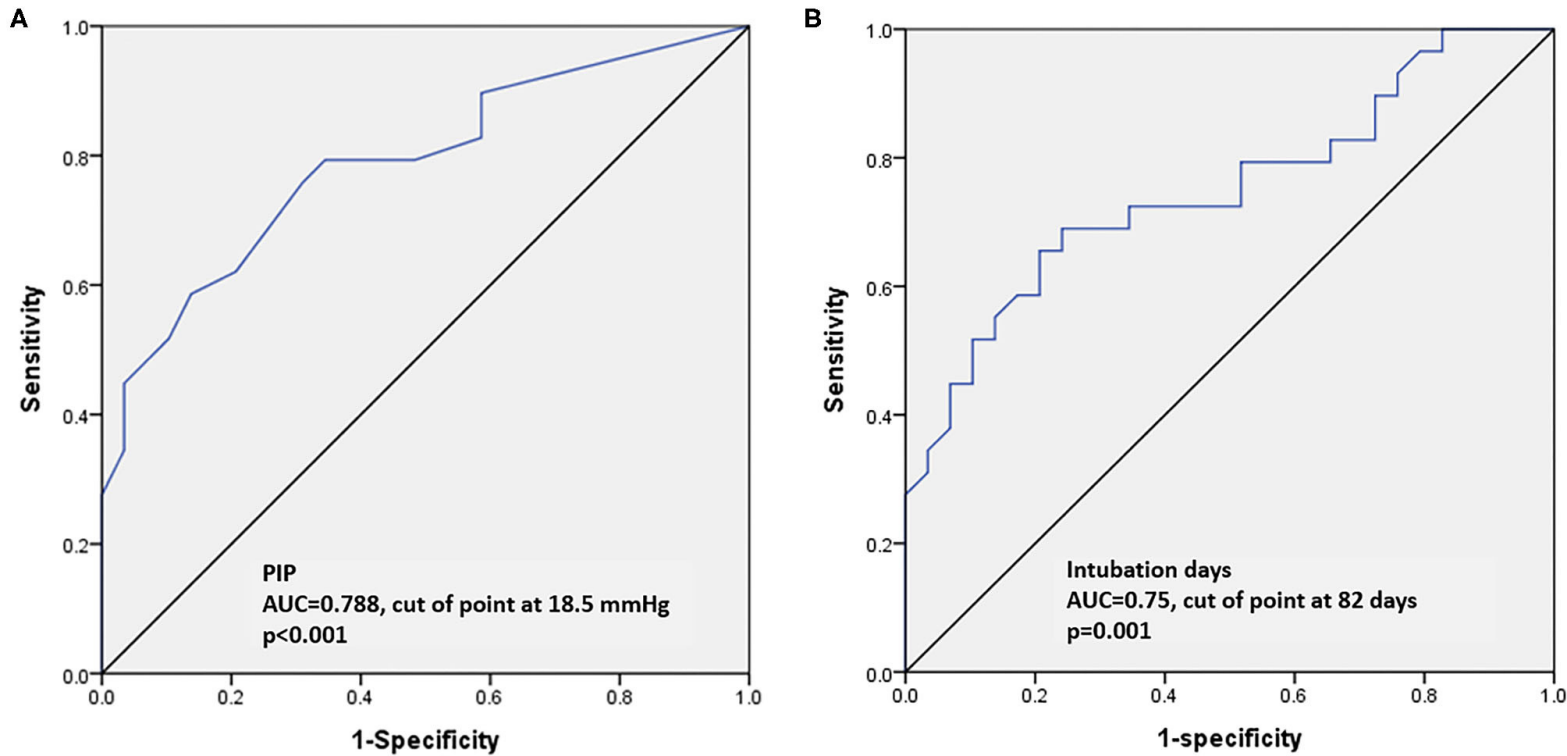
Receiver operating characteristic curves to assess the predictive accuracy of parameters for TBM. **(A)** Peak inspiratory pressure. **(B)** Duration of intubation (days).

Multivariate logistic regression analysis was performed to investigate the impact of multiple risk factors on the development of TBM in the preterm infants with BPD. Severe BPD, PIP in the week before the bronchoscopy examination and the duration of intubation remained significantly different between the patients with and without TBM in the final model (Table 2). Among these factors, severe BPD was the most powerful risk factor for the development of TBM (odds ratio 5.57, 95% confidence interval 1.32–23.5, *p* = 0.019). ROC curves were used to determine the optimal cut-off values for predicting the development of TBM. The areas under the ROC curves for PIP and the duration of intubation were 0.788 and 0.75, respectively (Figures 2A,B). The best predictive cutoff values of PIP and duration of intubation for TBM were 18.5 mmHg (sensitivity: 0.79, specificity: 0.66) and 82 days (sensitivity: 0.65, specificity: 0.8), respectively (Figures 2A,B).

**Table 2:** Multivariate logistic regression analysis to predict the development of TBM.

**Parameters**	**β**	**Odds Ratio (95% CI)**	***P*-value**
Severe bronchopulmonary dysplasia	1.72	5.57 (1.32–23.5)	0.019^*^
Average MAP during the first week (cmH_2_O)	0.15	1.17 (0.8–1.7)	0.412
**Average ventilator setting during**			
**the week before bronchoscopy**			
PIP (cmH_2_O)	0.08	1.092 (1.014–1.177)	0.02^*^
PEEP (cmH_2_O)	0.28	1.32 (0.76–2.29)	0.317
MAP (cmH_2_O)	−0.11	0.89 (0.58–1.38)	0.621
Duration of Intubation (days)	0.023	1.024 (1.001–0.147)	0.046^*^

*MAP, mean airway pressure; PIP, peak inspiratory pressure; PEEP, positive end expiratory pressure.*

* *P < 0.05 statistically significant*.

### Outcomes

Comparisons of in-hospital morbidity and mortality between the patients with and without TBM are shown in Table 3. The patients with TBM had a significantly longer hospital stay and more episodes of pneumonia during their hospital course. Furthermore, the patients with TBM had a higher in-hospital mortality rate compared to those without TBM.

**Table 3:** Comparisons of in-hospital morbidity and mortality between the infants with and without TBM.

**Outcomes**	**TBM (*n* = 29)**	**No TBM (*n* = 29)**	***P*-value**
Extubation failure (times), mean (SD)	1.8 ± 1.9	1.1 ± 1.4	0.1
Hospital stay (days), mean (SD)	198.8 ± 52.1	131.2 ± 38.6	<0.001^*^
Pneumonia (times), mean (SD)	1.8 ± 1.3	1.1 ± 1.4	0.016^*^
Tracheostomy, n (%)	5 (17.2)	4 (13.7)	0.72
GERD, n (%)	9 (31)	7 (24.1)	0.769
Gastrostomy, n (%)	2 (6.8)	2 (6.8)	1
Death, n (%)	6 (20.6)	1 (3.4)	0.043^*^

*GERD, gastroesophageal reflux disease.*

* *P <0.05 statistically significant*.

## Discussion

TBM is often underestimated in neonates with BPD. In a large multicenter cohort study of 974 neonates with BPD, Hysinger EB et al. reported that more than one-third of the cohort who underwent bronchoscopy had TBM (12). In the current study, we found a higher rate of TBM in our preterm infants with BPD (50%), which may be because we included infants with a younger gestational age than Hysinger's study (12). In the present study, we found that the infants with TBM were associated with higher rates of morbidity and mortality than those without TBM, which is consistent with Hysinger's study (12). The high incidence of TBM and its association with adverse outcomes emphasizes the need for better therapies for these preterm infants. Therefore, we investigated the risk factors for the presence of TBM, and found that severe BPD was the most powerful risk factor, and that PIP and the duration of intubation were also risk factors for the presence of TBM. Moreover, we determined the optimal cut-off values to predict the presence of TBM, which is valuable information that can help intensivists to adjust therapeutic strategies for these preterm infants.

In preterm infants, the immature tracheal cartilage is hypercellular with little glycosaminoglycans, and this results in a more compliant and smaller airway (1). The softness of the immature airway is vulnerable and easier to deform when exposed to positive pressure ventilation (1, 13). In preterm infants with BPD, the histopathologic changes of the airway include smooth muscle hypertrophy, thickening of airway walls, epithelial inflammation, and septal and parenchymal fibrosis (1, 13, 14), which can result in higher intrinsic positive end-expiratory pressure (PEEPi) and peripheral airway resistance (15), and subsequently an increase in collapsing transmural airway pressure (1). Clinically, severe BPD is associated with higher PEEPi and airway resistance than mild and moderate BPD, resulting in more severe airway collapsibility (15).

In the current study, we also demonstrated that higher airway pressure and prolonged duration of intubation were independent risk factors for the presence of TBM in the preterm infants. This may be because barotrauma caused by high positive pressure ventilation weakens tracheal and bronchial walls and predisposes them to collapse during expiration (10, 13–16). To maintain the airway patency, higher positive pressures are often used as a “pneumatic stent,” which consecutively exacerbates pressure-induced injury and creates a vicious circle resulting in constant airway damage in these infants (11, 17, 18). Among the different pressure settings, PIP has been reported to be the most powerful predictor for the presence of TBM. Previous studies have also demonstrated that prolonged intubation is a major risk factor for the development of TBM (19, 20). The cause of TBM following prolonged intubation is assumed to be multifactorial. First, prolonged intubation causes airway damage through ischemic necrosis in the contact area between the tube and the underlying tracheal wall (21, 22). In addition, frequent sputum suction and manual ventilation, combined with physiologic sucking and swallowing cause cyclic friction damage to the airway mucosa (23). Finally, infants who require prolonged intubation often have concomitant severe BPD. The structural heterogeneity of the lungs contributes to PEEPi formation, which increases the negative transmural pressure gradient and airway narrowing during expiration (11, 15). These mechanical injuries and pathophysiological changes result in chronic inflammation and exaggerate the weakness of the premature airways (24). In the current study, we identified two important risk factors associated with mechanical ventilation and their optimal cut-off levels for predicting the presence of TBM (PIP: 18.5 mmHg, duration of intubation: 82 days). Our results indicate that to prevent the development of TBM, clinicians should consider a ventilation strategy of protecting the developing lungs from mechanical ventilation. This strategy should include weaning from the ventilator as quickly as clinically possible (and avoid a PIP >18.5 cmH_2_O), and then early extubation (before 82 days) followed by non-invasive ventilatory support whenever possible.

In this study, the preterm infants with BPD and TBM were associated with more complicated adverse outcomes, including a trend of higher extubation failure, longer length of hospital stay, more episodes of pneumonia and higher in-hospital mortality rate, which is consistent with a previous study (12). Airway collapse during expiration is associated with recurrent respiratory infections in preterm infants, because an increase in sputum retention can result in poor airway hygiene (25). Furthermore, these preterm infants usually have more episodes of extubation failure and prolonged ventilatory support, which are associated with a prolonged length of hospital stay (14, 26, 27). Consequently, the major causes of in-hospital mortality are severe BPD accompanied by pulmonary hypertension and a poor response to medications.

### Limitations

The current study has several limitations. Only preterm infants with clinical symptoms who underwent bronchoscopy were included, which may have led to selection bias. In addition, there was no standardization of the indications or the timing of the bronchoscopies, and there was also heterogeneity of ventilatory strategies owing to the retrospective nature of the study. Future prospective studies with standardization of the indications for the bronchoscopies should be conducted to verify our results.

### Conclusion

In conclusion, preterm infants with severe BPD are at high risk for the presence of TBM. In addition, the risk is even higher in those who receive a higher PIP or are intubated for longer. Bronchoscopy examinations should be arranged for preterm infants with severe BPD, and for those who require a higher PIP and longer duration of intubation to allow for an early diagnosis and timely management of TBM.

## Data Availability Statement

The raw data supporting the conclusions of this article will be made available by the authors, without undue reservation.

## Ethics Statement

The studies involving human participants were reviewed and approved by the Ethics Committee of Chang Gung Memorial Hospital (approval no. 202000797B0). Written informed consent to participate in this study was provided by the participants' legal guardian/next of kin.

## Author Contributions

Y-TS, C-CC, S-HH, and E-PL conceived and designed the study. S-HL, C-YC, and P-LT collected data. C-CC, J-JL, and O-WC performed data analysis. E-PL interpreted the data. Y-TS drafted the manuscript. All authors approved the final version of the manuscript.

## Conflict of Interest

The authors declare that the research was conducted in the absence of any commercial or financial relationships that could be construed as a potential conflict of interest.
